# Melting line of calcium characterized by in situ LH-DAC XRD and first-principles calculations

**DOI:** 10.1038/s41598-021-94349-4

**Published:** 2021-07-22

**Authors:** Simone Anzellini, Dario Alfé, Monica Pozzo, Daniel Errandonea

**Affiliations:** 1 Diamond Light Source Ltd., Harwell Science & Innovation Campus, Diamond House, Didcot, OX11 0DE UK; 2 Dipartimento di Fisica Ettore Pancini, Università di Napoli Federico II, Monte S. Angelo, 80126 Napoli, Italy; 3 Department of Earth Sciences and London Centre for Nanotechnology, University College London, Gower Street, London, WC1E 6BT UK; 4 Departamento de Física Aplicada - Instituto de Ciencia de Materiales, Matter at High Pressure (MALTA) Consolider Team, Universidad de Valencia, Edificio de Investigación, C/Dr. Moliner 50, Burjassot, Valencia, 46100 Spain

**Keywords:** Materials science, Physics, Condensed-matter physics

## Abstract

In this work, the melting line of calcium has been characterized both experimentally, using synchrotron X-ray diffraction in laser-heated diamond-anvil cells, and theoretically, using first-principles calculations. In the investigated pressure and temperature range (pressure between 10 and 40 GPa and temperature between 300 and 3000 K) it was possible to observe the face-centred phase of calcium and to confirm (and characterize for the first time at these conditions) the presence of the body-centred cubic and the simple cubic phase of calcium. The melting points obtained with the two techniques are in excellent agreement. Furthermore, the present results agree with the only existing melting line of calcium obtained in laser-heated diamond anvil cells, using the speckle method as melting detection technique. They also confirm a flat slope of the melting line in the pressure range between 10 and 30 GPa. The flat melting curve is associated with the presence of the solid high-temperature body-centered cubic phase of calcium and to a small volume change between this phase and the liquid at melting. Reasons for the stabilization of the body-centered face at high-temperature conditions will be discussed.

## Introduction

The phase diagrams and melting behaviour of the alkaline-earth metals (group IIa in the periodic table) at extreme conditions of pressure (*P*) and temperature (*T*) are of fundamental interest in condensed matter physics and material science. In fact, the presence of a nearly empty *d*-band, lying in close proximity to the *sp*-valence band, strongly affect their behaviour at extreme conditions: showing relatively large compressibility and an unusual trend in going from highly symmetric (densely packed) structures to a more open-packed one^1,2^.

Among the alkaline-earth metals, calcium (Ca), attracts considerable interest due to its unexpectedly complex phase diagram, its loss of metallic properties under pressure^3^ and for being the element with the highest critical temperature (25 K) under high pressure^4,5^. For these reasons, the phase diagram and equation of state (EoS) of Ca have been extensively studied both experimentally^2,4–13^ and theoretically^3,11,14–16^ over the years.

However, most of the reported studies focus on the characterization of Ca under high *P* and room or low *T* conditions. Very few high *P*-*T* experiments have been performed on Ca^6,9,10,17^ in part due to its high chemical reactivity at high *T*. Among the high *P*–*T* studies on Ca, only a recent study by Anzellini et al.^6^ has used X-ray diffraction (XRD) to unambiguously determine the observed crystal structures. In this particular study, performed using a resistively-heated diamond anvil cell (DAC) coupled with synchrotron XRD, the high *P*–*T* phase diagram of Ca was characterized between ambient conditions and 53 GPa—800 K. In the reported *P*–*T* range, the face-centred (*fcc*, Ca-I), body-centred (*bcc*, Ca-II) and simple cubic (*sc*, Ca-III) phases of Ca were observed. In particular it was possible to establish a connection between the *bcc* phase previously observed at low *P* and high *T*, with the one reported at ambient *T* and high *P*. However, nothing is known about the stability of *bcc* Ca above 800 K.

Concerning the melting line of Ca, early experiments performed in a piston-cylinder apparatus reported a melting temperature of 1500 K at 4 GPa based on differential thermal analysis^9,10^. The only other information on the melting curve of calcium was obtained up to 80 GPa and 3000 K using a laser-heated DAC (LH-DAC)^8^. In this experiment, the pressures were determined ex situ using the ruby fluorescence method^18^ and the temperature was determined using spectral radiometry^19^. The melting of the sample surface was probed using the laser-speckle technique^20^. The obtained results showed an unusual trend of the melting line, with a slope flattening around 1500 K between 5 and 32 GPa, dramatically increasing between 32 and 45 GPa [(dT /dP) ~ 96 K/GPa] and flattening again around 45 GPa and 2750 K [(dT /dP) ~ 3 K/GPa]^8^. The observed behaviour suggested that below 32 GPa Ca melts from the Ca-II (*bcc*) phase and that there must be a Ca-II–Ca-III-liquid (*bcc*-*sc*-liquid) triple point located around 32 GPa and 1550 K.

The lack of structural information on the phase diagram of Ca above 800 K, has highlighted the need for the present study. In fact, with the use of LH-DAC coupled with angular-dispersive synchrotron XRD, it is possible to obtain an in situ characterization of phase diagrams and melting lines of samples at extreme *P*-*T* conditions (in excess of 3 Mbar and 5000 K, respectively). ”Time resolved” analysis of the structural, chemical and textural evolution of the sample can be performed as a function of *P* and *T*. Furthermore, the nature of the XRD technique provides an objective melting criterion, i.e. the appearance of a diffuse halo in the diffraction pattern due to the scattering from the liquid sample. Such a characteristic is of great importance, as the only information on the melting line of Ca was provided using the speckle technique. As for some materials the validity of this method has been recently put into question^21–27^, it is fundamental to characterize the flattening of the melting curve of Ca with a more reliable technique. Furthermore, the combination of the obtained experimental results with first principle calculations, provides important insights on the dynamics of the observed phase-transitions.

**Figure 1:**
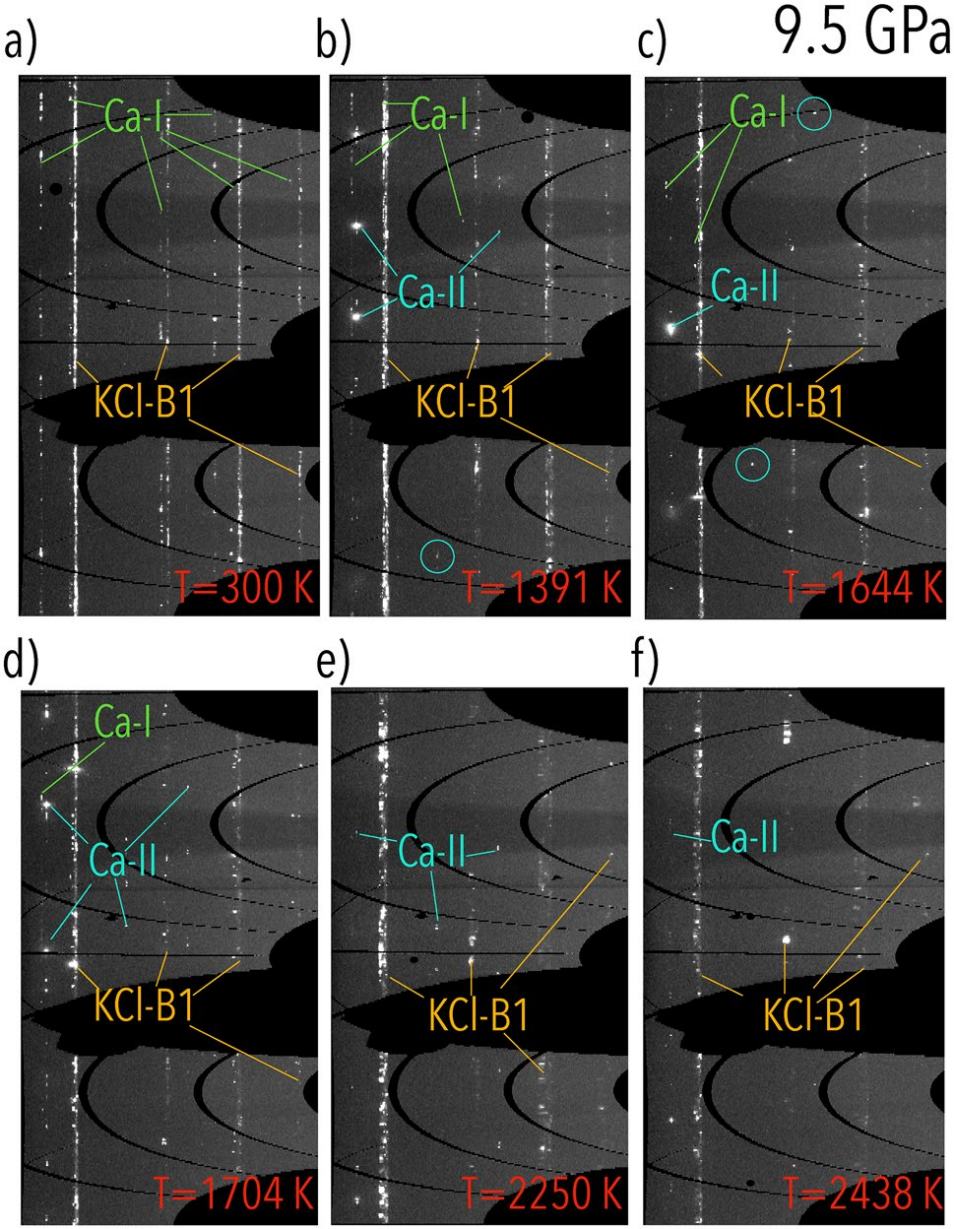
Textural evolution of a Ca sample embedded in KCl pressure transmitting medium at around 9.5 GPa. The different phases/samples observed in the patterns are highlighted with different colours: green for Ca-I, light blue for Ca-II and orange for KCl-B1.

## Results

### Experiments

Five experimental runs have been performed at Diamond Light Source (DLS), using the experimental conditions reported in the “Methods” section. Each run was performed on a different region of the Ca samples, so to minimize the presence of any possible chemical reactions obtained in previous measurements. During the experiments, it was possible to cover a *P*–*T* range between 10 and 40 GPa and between ambient *T* and 3000 K, respectively. In this investigated portion of the phase diagram, only the I, II, and III solid phases of Ca and the B2 phase of KCl were observed (in addition to liquid Ca). In one experiment performed near 40 GPa in Ca-III, the presence of a weak signal from the B1 phase of CaO was detected and refined according to the parameter reported in Richet et al.^28^. This suggests that Ca-III could be more reactive to oxygen than Ca-I and Ca-II. However, the presence of this partial chemical reaction did not affect the present measurements.

**Figure 2:**
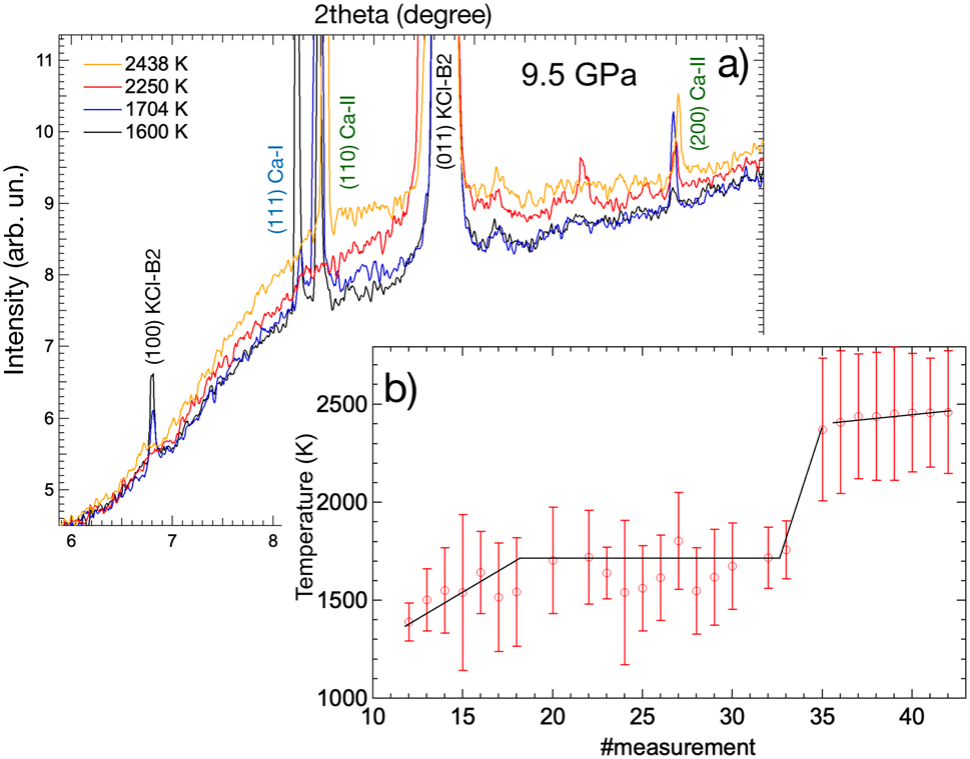
(**a**) Integrated XRD patterns at different temperatures at 9.5 GPa. The onset of melting, characterized by the first appearance of diffuse signal, is observed at 1704 K (blue pattern). The diffuse scattering increases with the rising temperature, up to a maximum of 2438 K (orange pattern), where most of the sample is melted. (**b**) Evolution of the average *T* during the ramp. The black lines have been drawn as guides for the eyes.

Figure 1 shows the textural evolution observed in the run carried out around 9.5 GPa from room *T* up to 2438 K. At ambient *T* (Fig. 1a), the XRD signal of Ca has the texture of an oriented powder corresponding to Ca-I. The observed orientation in the grains at ambient *T* is caused by the previous alignment of the lasers with the sample. Around 1391 K, together with Ca-I, it is possible to observe the presence of a single-crystal like signal from the Ca-II phase. The presence of both Ca phases at this *T* conditions is linked to the axial thermal gradients (around 800 K) developed between the two sample surfaces and caused by both the nature of the LH-DAC technique^29^ and the adopted sample loading. The presence of this thermal gradient is further proved by the different textural behaviour observed in the two phases. In fact, while Ca-I shows only a partial re-crystallization between ambient temperature and 1704 K (Fig. 1a–d), Ca-II immediately has a single-crystal-like texture showing a ”fast re-crystallization”^23,30,31^ phenomenon with the temperature increase. Furthermore, at 1704 K, it is detected the onset of the solid-liquid transition (Fig. 2), both phases are still present and start disappearing only at higher *T* when a large portion of the sample is melted (Fig. 1e,f).

Figure 2a, shows the corresponding evolution of the integrated diffraction signal for the same ramp. The signal at 1704 K (in blue) starts showing a clear signal of diffuse scattering coexisting with Bragg peaks from Ca-II. As previously discussed, it is possible to observe how the diffuse scattering is enhanced with the increasing *T*, as shown in the pattern at 2250 K (in red) and at 2438 K (in orange). Such an increase in the diffuse scattering is an indication that a larger part of the sample is liquid. A plateau was also observed in the measured *T* around 1700 K. This can be seen in Fig. 2b. Plateaus in the *T* distributions are generally correlated with the occurrence of phase transitions. In this case the simultaneous observation of the *T* plateau and the diffuse scattering is undoubtedly supporting the melting of Ca. The measured thermal evolution also show the formation of a second plateau at higher *T*. However, this is observed at *T* corresponding to the melting *T* of the insulating material (KCl) at the same *P* conditions^32^, therefore the second plateau must be related to the melting of the portion of KCl in contact with the sample.

Similar results have been observed in the other ramps. At 13 and 16 GPa the samples, originally in the Ca-I phase at ambient *T* transformed to the Ca-II phase above 1200 K (the lowest *T* measurable with the present setup) to be molten above 1700 K. At 25 GPa, the sample maintained its Ca-II phase from room *T* up to 1700 K, where it melted. Finally, at 40 GPa the sample presented a Ca-III phase in the investigated *T* range up to the measured melting *T* of 2000 K. In this case, a partial formation of CaO was clear, suggesting a more enhanced reactivity of the Ca-III with respect to the other two solid phases. This is consistent with the well-known pressure-induced *sp*-*d* electron transfer in Ca^1–3^. Normally, the oxidation of Ca is expected to occur only on the sample surfaces^33^. Since the melting is detected from XRD produced by X-rays going through the entire sample, and it is detected at *T* much lower than the melting *T* of CaO^34^, the presence of partial formation of the latter is not expected to affect the present characterization.

**Figure 3:**
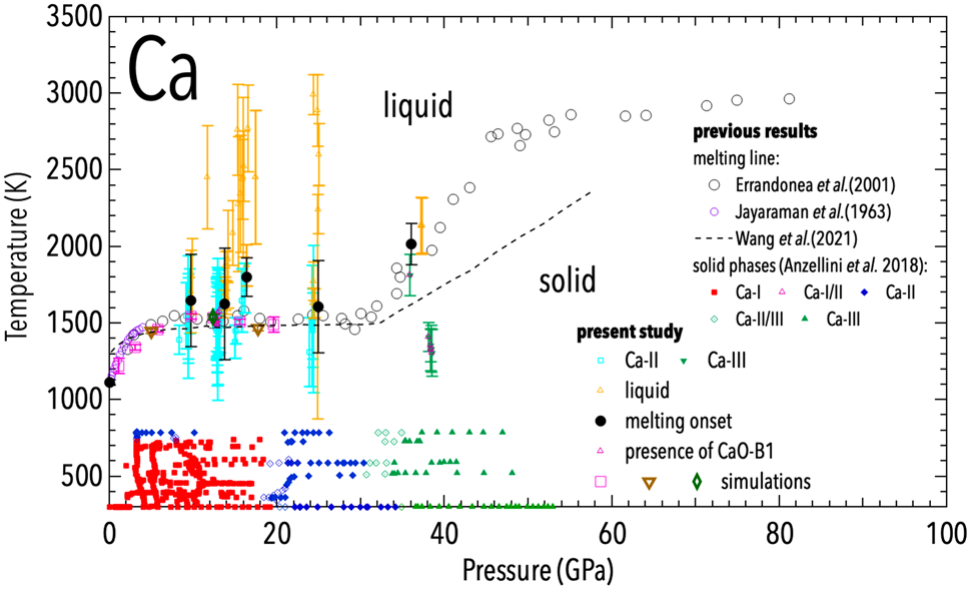
Phase diagram of Ca as measured (light blue, green and gold symbols) and simulated (empty purple squares, brown triangles and green diamond symbols) in the present study, compared to previous experimental results obtained in DAC from resistive^6^ and laser-heating^8^ experiments and in piston-cylinder apparatus^9^. Results obtained from ab initio molecular dynamic simulations are also reported for comparison^36^.

Concerning the accurate measurement of the melting *T*, it is often discussed how it could be overestimated by experiments performed using LH-DAC combined with XRD^25,31,35^. For this reason, although the *T* of the last solid and first liquid found in this study are quite close in values and within the experimental errors, the actual melting *T* have been considered as the average between those two values and are reported in Fig. 3 as black circles.

Considering the thermal evolution of the lattice parameters, the results obtained around 9 GPa (1400 K $< T$ < 1600 K) lead to an average linear expansion coefficient of 7(1) $\times$ 10$^{-5}$ K$^{-1}$. This agrees, within the experimental errors, with the value of the thermal EoS reported in Anzellini et al.^6^ using a Berman model for temperatures below 800 K. However, according to the present study, at higher pressures (15 GPa and 24 GPa), the value of the linear expansion coefficient is drastically reduced to 1.5(9) $\times$ 10$^{-5}$ K$^{-1}$. This suggests that, in the case of Ca, the thermal expansion is strongly affected by *P*. Such an effect is not taken into account by the EoS adopted in Anzellini et al.^6^, which assumes only a *T* dependence of the thermal expansion. Although such a model works well for many metals^25,37,38^, that is not the case for Ca.

According to the present experiment, the unit-cell volume of Ca-II at the melting *T* (1500 (200) K) is 57.9(5) ${\AA }^3$ at 15 GPa and 49.1(5) ${\AA }^3$ at 24 GPa. These values are approximately 10% smaller than the values obtained using the *PVT* EoS determined by Anzellini et al.^6^ with data obtained up to 800 K. This suggests that anharmonic effects, not considered in the Berman model, are not negligible in Ca for *T* close to melting.

### Calculations

**Figure 4:**
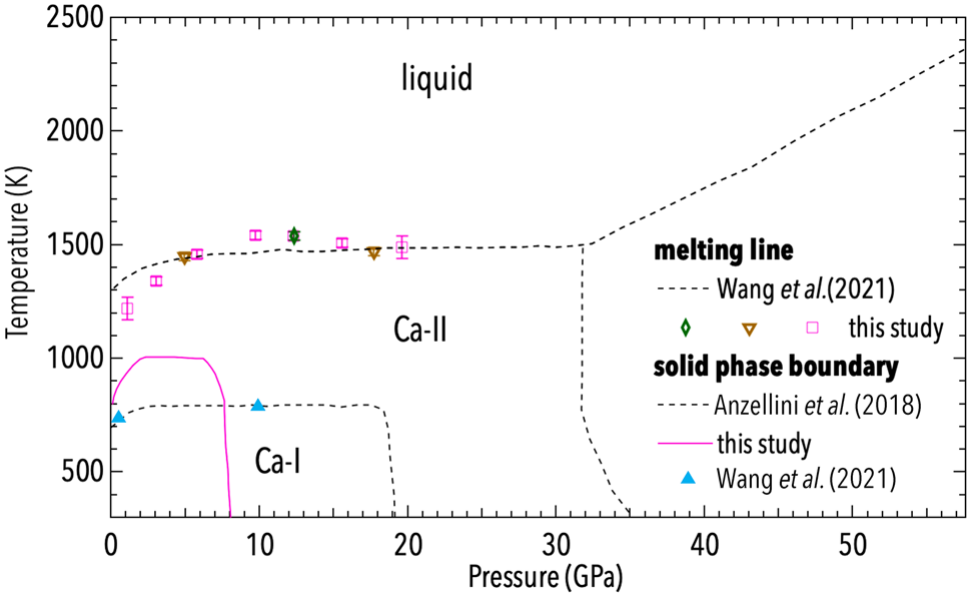
Phase diagram of Ca as obtained with the present first principle calculation (empty symbols and pink continuous line) and a previous study based on ab initio molecular dynamics simulation^36^ (dashed black line and light blue triangle) and a high *P*-*T* experiment (dashed black lines)^6^. The different symbols used in the graph represent the three different approaches used for the simulations. In particular: the pink squares represent the simulation obtained with 2 valence electrons and 1000 atoms; the brown triangles are obtained using 10 valence electrons and 1000 atoms; the green diamond are obtained using 2 valence electrons and 1960 atoms.

In Fig. 4 the results from the present calculations are summarized. In particular, it is possible to observe the calculated Ca-I/Ca-II phase boundary and melting temperatures (from the Ca-II phase) at different pressures.

Similarly to the simulations obtained by Wang et al.^36^, the present calculations reproduce qualitatively the experimental phase boundary reported by Anzellini et al.^6^. However, they seem to underestimate the Ca-I/Ca-II transition pressure. In particular, the obtained Ca-I/Ca-II transition *T* increases as a function of *P* from ambient pressure up to 3 GPa. Above this *P*, the *T* of the phase transition becomes *P*-independent up to 7 GPa, when it suddenly decreases with a nearly vertical slope.

This strong non-linear dependence of the phase boundary is caused by two phenomena, which favour the stabilization of the Ca-II phase. In particular, at high *T* the Ca-II structure is favoured by entropy effects^39^, whereas at high *P* this structure becomes favoured because of the *sp*-to-*d* electron transfer^40^. The positive slope at low *P* is due to the fact that the Ca-II phase at high *T* has a larger volume and entropy than the Ca-I. Then, according to the Clausius-Clapeyron equation ($dT/dP = \Delta V/ \Delta S$), the slope of the phase boundary should be positive as determined by the present calculations. However, at high *T* the Ca-II phase is more compressible than the Ca-I phase, becoming $\Delta V = 0$ at 3 GPa. This could explain why at this conditions, the Ca-I/Ca-II transition *T* becomes *P*-independent. Upon further compression, the reported solid-solid phase boundary (with a negative slope) is driven by the *sp*-*d* electron delocalization, producing a large volume collapse. Therefore while $\Delta S > 0$ as at high *T*, $\Delta V < 0$. This explains the negative slope of the phase boundary. On the other hand, the large volume and small entropy change is what makes the phase boundary nearly vertical.

## Discussion

In Fig. 3, the present experimental and first principle results are compared with the ones obtained in previous studies. Both the present experiments and calculations are in excellent agreement between each other, and are supporting the melting of Ca from the Ca-II phase below 25 GPa. This is in agreement with the Ca-II phase reported at high *T* in the same *P* range from the resistively-heated experiments^6^. The present results also agree with previous studies where melting was determined using the speckle method^8^ and calculated via ab initio molecular dynamic simulations^36^. All those methods found that Ca has a flat melting curve from 5 to 30 GPa, being the melting *T* basically not affected by *P* in this pressure range. This fact is quite unusual in metals and can only be explained by the fact that above 5 GPa there is basically no volume change between Ca-II and liquid Ca.

From the present experiment, it was possible to determine the unit-cell volume of solid Ca-II just below the melting *T* and to characterize its *P*-induced evolution. As previously discussed, the volume of Ca-II changes at 1500 K (the melting *T*) from 57.9 (5) ${\AA }^3$ at 15 GPa to 49.1 (5) ${\AA }^3$ at 24 GPa (relative change of 13%). Therefore, a similar change must be expected for the liquid Ca (at these *P*-*T* conditions), being such a *P*-induced change in the volume of liquid Ca comparable to those observed in liquid Co and Ni^41^ under a similar *P* change.

The present results for the Ca-III phase are also in good agreement with previous speckle method studies. They confirm that at the Ca-II–III–liquid triple point there is a large increase in the melting slope. The large positive melting slope of Ca-III indicates that there is an increase in the volume from Ca-III to liquid Ca at melting. This observation is in agreement with the fact that liquid Ca has a similar volume than Ca-II, but Ca-III has a much smaller volume due to the 20% volume collapse occurring at the Ca-II–Ca-III transition.

In conclusion, the results obtained in the present study provide for the first time a direct observation of melting in Ca at high *P*–*T* conditions. The obtained experimental and theoretical results are in excellent agreement and the performed first principle calculations provide an explanation to the fact that Ca-I transforms into Ca-II both at high *T* and at high *P*. Concerning the melting line of Ca, both experiments and calculations confirm the results obtained in previous studies, showing that Ca has an unusual flat melting curve below 30 GPa, a distinctive and unique feature of this metal. The present results also allow the determination of melting from the Ca-III phase to be obtained, showing that the Ca-II–III transition, and the associated volume collapse, deeply affects the melting curve of Ca.

## Methods

### Experimental

Three membrane diamond anvil cells (DAC) were equipped with diamonds with culet sizes ranging from 400 to 500 $\upmu$m. The gaskets where prepared from pre-indented and sparkle-erosion drilled Re foils. Sample loadings were performed under an inert atmosphere to prevent sample oxidation or other possible chemical reactions. Following the procedure described in Anzellini et al.^6^, samples were cut from a piece of 99% purity Ca, sourced from Goodfellow, with Al (800 ppm) and Mg (1000 ppm) as main impurities (according to the vendor spreadsheet). The obtained samples were squeezed between two FIB-cut KCl disks and loaded in the DAC’s high-pressure chambers. The KCl disks, oven dried at 200 $^\circ$C for two hours before being loaded in the DAC, were used as both insulating material (thermal and chemical) as well as pressure gauge.

The experiment was performed at the extreme condition beamline I15 of the Diamond Light Source (DLS) synchrotron^42^. I15’s polychromatic beam was tuned to 29.3 keV and focussed down to 6 × 9 $\upmu$m$^2$. A Pilatus 2M detector was used to ensure fast data collections with a good signal/noise ratio. The sample-to-detector distance was measured following standard procedure from the diffraction rings of a CeO$_2$ standard.

The high *P*–*T* experiment were performed using the beamline LH-system^42^, following the procedure described in Anzellini et al.^25^. Before each heating ramp, the sample was brought to the target pressure. The *P* was measured from the compression curve of the KCl according to the thermal EoS of Dewaele et al.^43^. Two 100 W Nd:YAG fibre lasers were individually focused on both sample surfaces. During the experiment, the *T* of the sample was measured by spectral radiometry (between 450 nm and 950 nm) following the procedure described in Anzellini et al.^29^. *T* were collected from both sides (upstream and downstream) of the sample and the average between those two readings was taken as the actual *T* value. The error in each *T* measurement was assumed to be the maximum value between the difference in the upstream and downstream *T* and the full width at half maximum (FWHM) of their histograms obtained from the two-colours pyrometry (as described in Benedetti and Loubeyre^19^). The corresponding thermal *P* was measured from the EoS of Dewaele et al.^43^ under the assumption that KCl and Ca were at the same *T*. Considering the sample loading geometry and the corresponding axial thermal gradients^29^, it is possible to estimate the maximum error in *P* varying from 1.2 GPa at 1200 K and 3 GPa at the maximum *T* reached^25^. In order to maximize the size of the hot spot at uniform *T* the two lasers were unfocused towards the sample. Once coupled together, their relative positions were adjusted to obtain a uniform hot spot over about 40 $\upmu$m in diameter. Before and after each heating ramp the relative alignment of the X-rays with the lasers and the temperature reading was checked following the procedure described in Anzellini et al.^42^.

The heating ramps were performed in ”trigger mode” i.e. both lasers were set to a target power, after 0.3 s a diffraction pattern and a temperature measurement were collected simultaneously. Then, 0.3 s after the XRD collection, both lasers were turned off. Such a procedure enable the minimization of the interaction time between the lasers and the sample (reducing the probability of possible chemical reactions). Plus, as each *P*–*T* point is individually collected, it is possible to adjust the alignment of the experimental setup during the ramp, before spoiling the sample conditions.

During each ramp, the laser’s target powers were increased until a diffuse ring (characteristic of a liquid sample) was detected on the diffraction pattern, or a hole was laser-drilled in the sample. Several heating-ramps were performed at different *P* for each sample. In order to avoid any chemical contamination, each ramp was performed on a different region of the sample. The quality of the selected region was checked by XRD before the heating started. An accurate analysis of the diffraction patterns was performed in order to detect the appearance of the melting and to obtain structural and textural information about the sample and the insulating material.

During the analysis procedure, masks were applied on a per-image basis using the DIOPTAS suite^44^. The images were azimuthally integrated and a Le Bail analysis was performed using the TOPAS suite^45^ using previously reported parameters^6^ as starting values. The structural measurements were compared to the *T* ones in order to obtain a detailed *in situ* and ”time resolved” analysis of the sample evolution as a function of *P* and *T*.

### Theoretical

The calculations were based on finite temperature density functional theory (DFT)^46–48^ using the exchange-correlation functional known as PBE^49^, as implemented in the vasp code^50^. The projector-augmented-wave (PAW) formalism^51,52^ was used and, for the majority of the calculations, a Ca PAW potential with an [Ar] core (2 electrons in valence) and an outmost cutoff radius of 1.96 Å, was adopted. Single particle wave functions were expanded in plane-waves (PW), with a cutoff of 103 eV. During the process, the obtained results were spot-checked using a PAW potential with a [Ne] core (10 electrons in valence), using an outmost cutoff radius of 1.22 Å and a PW cutoff of 267 eV.

The phase diagram has been obtained using a combination of harmonic calculations for the solid region, and direct solid–liquid coexistence to determine the melting points. The harmonic calculations have been performed on the *fcc* and the *bcc* phases of Ca, using the small displacement method as implemented in the phon code^53^. A $4 \times 4 \times 4$ supercell (64 atoms) and a $4\times 4\times 4$ grid of **k**-points were used to sample the Brillouin zone for both structures.

The melting curve was computed using the coexistence method^54^. With this method, solid and liquid are simulated side by side in a large box, using molecular dynamics (MD). If the simulations are done at constant volume and constant internal energy (microcanonical ensemble, or NVE), then there is a whole range of internal energies for which coexistence between solid and liquid is maintained^55^. The average *P* and *T* over the MD run provides a point on the melting curve.

As the solid part of the phase diagram shows that the *bcc* phase is the most stable at high *T*, the melting calculations have been performed assuming coexistence with this phase. As an independent cross check, the melting *T* from the *fcc* phase was also computed at one *P*, and found to be lower than the corresponding melting *T* for the *bcc* phase, confirming the thermodynamic stability of this latter phase.

In order to prepare the system to determine melting from the *bcc* phase, a ($5 \times 5 \times 20$) supercell (1000 atoms) was used. This was initially equilibrated at a guessed melting *T* for 2000 steps, each with a length of 0.5 fs. The equilibrium condition was obtained by performing MD simulations at constant *T* using a combination of Nosé^56^ and Andersen thermostats^57^ and sampling the Brillouin zone with the $\Gamma$ point only. Once the equilibrium was reached, the simulation was stopped and the atoms in one half of the simulation cell were clamped, whereas those in the other half were left free to move at a *T* about 10 times higher than the original one. This simulation continued until good diffusion was observed for at least 1 ps. After that, the simulation was stopped again and the system was simulated for an additional 1 ps at the guessed melting *T*, still holding the atoms in the solid part of the cell clamped.

**Figure 5:**
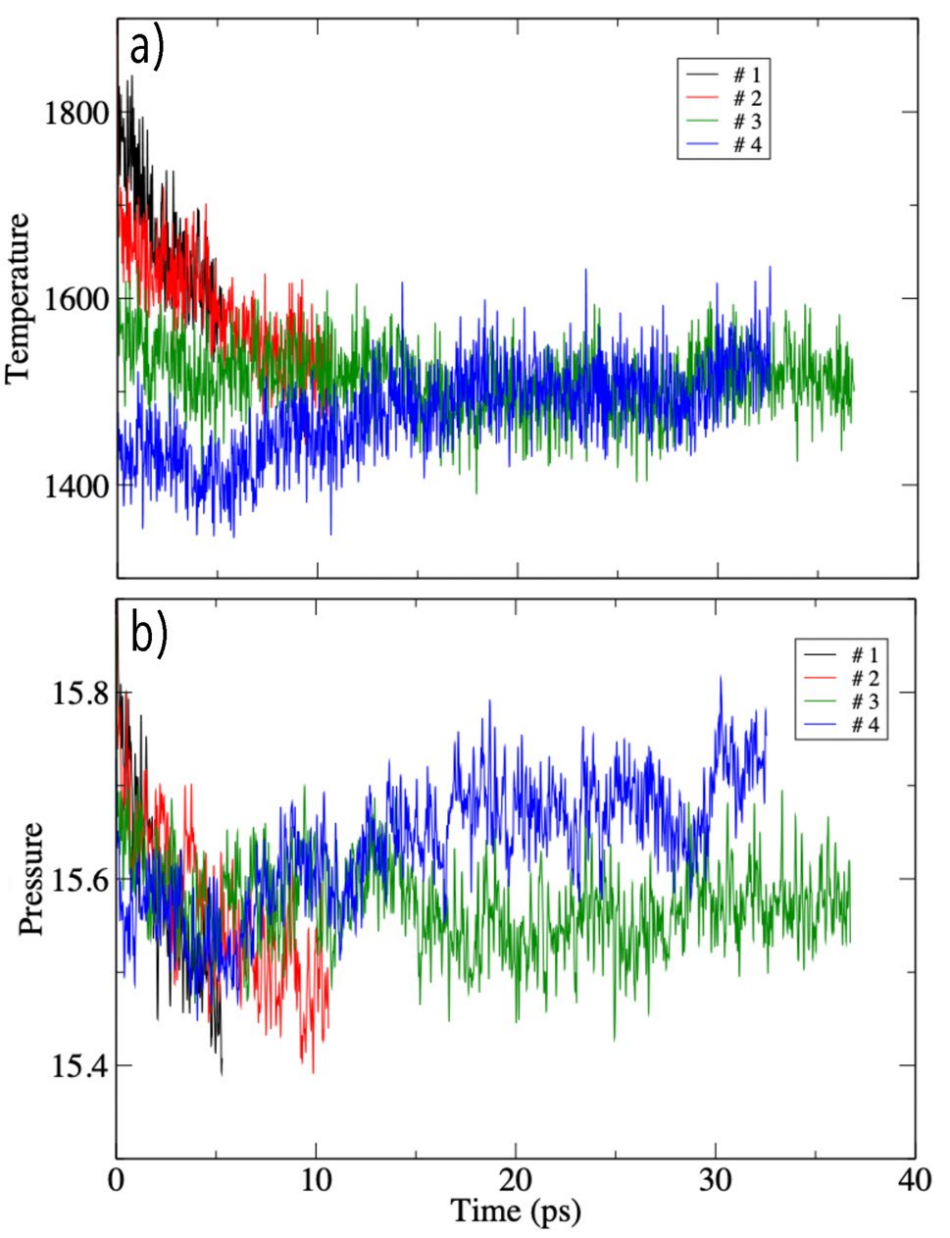
Instantaneous (**a**) temperature and (**b**) pressure of four solid-liquid coexistence simulations with different amount of internal energy. Simulation # 1 and # 2 both melt after ~ 5 and ~ 10 ps, respectively. In simulations # 3 and #4 solid and liquid coexist for the whole duration of the run.

After this preparatory cycle, all atoms are released, random initial velocities (drawn from a Maxwellian distribution) are assigned to them, and the system is simulated in the NVE ensemble, with *P* and *T* obtained as time averages as the simulation proceeds. The system is monitored by calculation of the average number density in slices of the cell taken parallel to the boundary between solid and liquid and by directly inspecting the positions of the atoms during the course of the simulations. The density in the solid part is a periodic function of slice number, while in the liquid it fluctuates rather weakly about its average value. A feedback mechanism is naturally built in the procedure: if *T* is higher than the melting *T* some of the solid will melt, absorb latent heat in the process and reduce *T*. If there is enough solid in the simulation cell the system stabilises with a new amount of solid and liquid, coexistence is maintained and a melting point can be obtained by averaging *P* and *T* over the equilibrated part of the simulation. If instead, the initial amount of solid is not enough, the whole solid will melt and the simulation cannot be used to obtain a melting point. This is the case in which the internal energy is outside (too high) the range of coexistence, and the simulation must be restarted with lower values for the initial velocities. Similarly, if the initial energy is too low the system will solidify, and the simulation needs to be restarted also in this case. The procedure therefore often involves a number of trial and error steps. As an example, *P* and *T* obtained for a number of simulations performed at $V/N = 28$ Å$^3$ are shown in Fig. 5. In simulation # 1 the amount of total energy is too high, and the system melts completely in less than 5 ps. Simulation # 2 is started with a lower amount of *E*, but this is still just outside the range of stability and the system also melts completely, but this time after about 10 ps. Note how in both simulation # 1 and # 2 *T* drops as the solid melts and absorbs latent heat in the process. Simulations # 3 and # 4 coexist for a long time, after an initial equilibration time during which the amount of solid and liquid in the simulation cell adjusts to the respective internal energy values. Since there is less energy in simulation # 4 than in # 3, system # 4 ends up with more solid, which is apparent in a slightly larger pressure. This also shows that at these conditions the volume change on melting is slightly negative, which is consistent with the slightly negative slope of the melting curve. All simulations at other thermodynamic conditions were continued for 35–50 ps to obtain melting points. The error on the melting temperature and pressure was calculated by standard re-blocking procedure^58^.

To test for size effects, one simulation was repeated using a ($7 \times 7 \times 20$) supercell (1960 atoms), from which the obtained results were compatible with those obtained with the smaller 1000-atom cells. The calculations were also repeated at two different volumes using the [Ne] core PAW potential. Because of the cost of the simulation with this PAW, these two simulations were only continued for about 10 ps. No noticeable drift was found in the instantaneous *P* and *T*, showing that the amount of solid and liquid is roughly constant throughout each simulation, which therefore can be used to extract a point on the melting curve even though they are relatively short. The points obtained using this harder PAW potential agree well with those obtained with the less expensive [Ar] core PAW potential.

Finally, we note that a coexistence simulation in the NVE ensemble can result in the build up of a non-hydrostatic *P*, due to the different volume of solid and liquid. In previous work^55,59^ this non-hydrostatic behaviour has been found to be very small and, by repeating the simulations at constant stress, it was found to have an undetectable effect on the calculated melting *T*. Here it was not possible to detect any non-hydrostatic behaviour (see Fig. 1 of Supplementary Materials), therefore the present NVE simulations are expected to be representative of hydrostatic behaviour.

## Supplementary Information

Supplementary Information.

## Data Availability

The datasets generated during and/or analysed during the current study are available from the corresponding author on reasonable request.
