# Supplementary material for: Melting line of calcium characterized by in situ LH-DAC XRD and first-principles calculations
Source: Sci Rep. 2021 Jul 22;11:15025. doi: 10.1038/s41598-021-94349-4 (PMC8298416; doi:10.1038/s41598-021-94349-4)
Supplement: Supplementary file 1 — Supplementary Information. [file 41598_2021_94349_MOESM1_ESM.pdf]

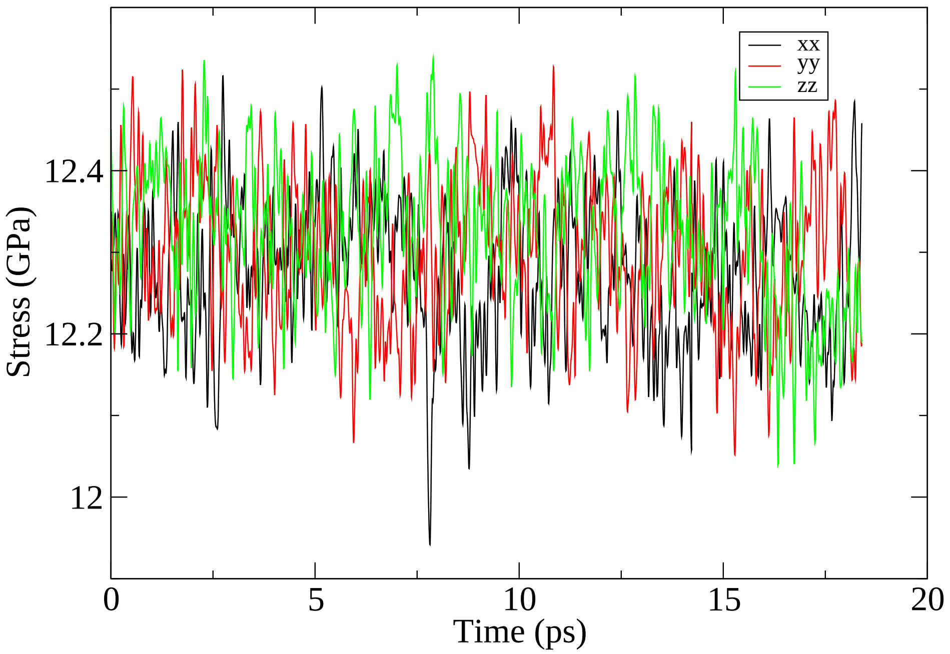

Figure 1. Diagonal stress components in a solid/liquid coexistence simulation performed with 1960 atoms, which gives a melting point $(p, T) = (12.3 \pm 0.2, 1540 \pm 20$ K)

.
